# Features of eye movements during rapid automatized naming in Chinese children aged 7–11 years

**DOI:** 10.3389/fped.2022.1051432

**Published:** 2022-11-29

**Authors:** Hongan Wang, Fulin Liu, Yuhong Dong, Dongchuan Yu

**Affiliations:** ^1^ Key Laboratory of Child Development and Learning Science of Ministry of Education, School of Biological Science and Medical Engineering, Southeast University, Nanjing, China; ^2^ Henan Provincial Medical Key Lab of Language Rehabilitation for Children, Sanmenxia Center Hospital, Sanmenxia, China; ^3^ Henan Provincial Medical Key Lab of Child Developmental Behavior and Learning, The Third Affiliated Hospital of Zhengzhou University, Zhengzhou, China

**Keywords:** developmental dyslexia, rapid automatized naming, eye tracking, school - aged children, reading abilities

## Abstract

Rapid Automatized Naming (RAN) tests have been well-documented to predict reading abilities as well as a variety of neurobiological disorders (e.g., developmental dyslexia). Traditional measures of RAN tests only take into account the naming time and accuracy and cannot reflect temporal-spatial features during RAN tests. Although the eye tracking approach appears to be a promising tool for characterizing the essential temporal-spatial characteristics of RAN tests, no research has been conducted to investigate whether and how gender, age, and task-type alter those characteristics. Additionally, no study has examined eye movements during a Chinese adaptation of RAN in order to expand the applicability of RAN to developmental dyslexia in Chinese. To address the concerns stated above, this article recruited 408 children (206 males, aged 7–11 years) and adopted eight measures to quantify features of eye movements during a Chinese adaptation of RAN. Findings showed that: (1) eight eye-movement measures had the main effects of task-type and age, but only five of them had the main effect of gender (in particular, females outperformed males); (2) RAN abilities observed by eight eye-movement measures initially developed quickly before the age of 9, and then entered a relatively sluggish development phase; (3) non-alphanumeric RAN tasks generally required higher mental load (implying more fixation counts, saccade counts, and regression counts, smaller average saccade amplitude, fixation duration fluctuation and saccade amplitude fluctuation, and longer average fixation duration and total time of naming) than alphanumeric ones; (4) there were significant correlations between total time of naming (a widely-used behavioral parameter) and other eye-movement measures; and (5) there were significant correlation between eight eye-movement measures and three attention-related skills observed from a number cancellation task. The current study might offer some perspectives on the understanding of normative data of eye movements during RAN in Chinese school-aged children, as well as the applications (e.g., developmental dyslexia) associated with RAN.

## Introduction

Rapid automatized naming (RAN) tests (1–12) have been proposed to evaluate an individual's ability to read letters, digits, objects, or other visual stimuli such as colors or geometric shapes as quickly as possible. These RAN tasks were initially used in studies on reading behavior and child development in healthy children, but they have gradually become recognized as a popular and useful psychometric test for identifying a variety of cognitive and neurobiological abnormalities, including developmental dyslexia (13–15), specific language impairment (16), attention deficit/hyperactivity disorder (ADHD) (17), learning disabilities, and autism spectrum disorder (ASD) (18, 19). In particular, findings (16, 20–24) have demonstrated that the RAN deficits may more accurately describe the characteristics of developmental dyslexia than other cognitive skill deficits. The goal of the current study was to offer some new perspectives on how to understand RAN more fully.

Traditional measures of RAN tests only take into account the naming time and accuracy and cannot reflect dynamical temporal-spatial features during RAN tests. While eye tracking techniques can be used to monitor the focus points in sequence and record the essential ocular activities throughout visual cognitive processes. Therefore, eye tracking approach would be a promising tool to characterize the visual cognitive features of RAN. Only a small number of studies (19, 25) have explored the characteristics of eye movements during RAN tests, but none of them have examined whether and how gender, age, and task type alter those characteristics. Additionally, no study has been done to analyze eye movements during a Chinese adaptation of RAN in order to expand the applicability of RAN to developmental dyslexia in Chinese. This study sought to identify the patterns or characteristics of Chinese school-aged children's eye movements during a Chinese adaptation of RAN (including naming Chinese characters). It should be noted that normative data of eye movement during RAN must be established in order to identify developmental abnormalities connected to RAN from eye movement data. However, thus far, no study has been conducted to establish normative data of eye movements during RAN in school-aged children, especially in China.

Taken together, we used the eye tracking technique to measure the characteristics of RAN eye movements and identified RAN eye movement characteristics in Chinese children aged 7–11 years. We specifically examined the characteristics of eye movements in Chinese children between the ages of 7 and 11 while they named Chinese characters. This is the first time, as far as we are aware, that eye movement characteristics during RAN testing for Chinese children aged 7–11 have been reported. In order to determine how the gender, age, and task type affect the characteristics of eye movements during RAN, we recruited 408 kids (206 of them were boys, ages 7–11). The relationship between eye-movement characteristics was examined. It was also explored how features of eye movements connect to abilities associated with attention.

## Materials and methods

The Southeast University Research Ethics Committee gave its approval to all study protocols and research techniques, ensuring that they adhered to the World Medical Association's Declaration of Helsinki regarding the use of humans in testing. All participating children's parents gave their informed consent, and each participant gave their verbal consent. After finishing the research, each kid was given a toy that was appropriate for their age.

### Study design and participants

The current study was conducted in Sanmenxia, Henan Province, China, between September 2021 to March 2022. According to the districts’ rankings of GDP per person in 2020, the districts of Sanmenxia were divided into three levels, i.e., strong economic level (>90,000 RMB), medium economic level (70,000–90,000 RMB), weak economic level (<70,000 RMB). In order to prevent bias in sample selection, we randomly selected a district with medium economic level and randomly selected an ordinary primary school locally from the district. This primary school included 1,387 kids (aged 7–11). According to the sequence number chosen randomly, a coding number was given to each child who was recruited. We only invited kids whose coding numbers with 3, 6 or 9 in the ones digit were to participate in our experiments.

Exclusion criteria were as follows: (a) abnormal hearing functioning (i.e., hearing threshold levels bigger than 25 dB HL) and vision functioning (i.e., naked or corrected monocular visual acuities below than 1.0); (b) significant sensory or motor impairment; (c) a history of previous neurological or psychiatric disorders; (d) IQ score lower than 85 or bigger than 115; (e) children who had repeated a grade; and (f) incomplete measure data.

By steps above, a total of 408 children (206 males) attended the current experiments (see Table 1 for detailed information). This survey complied with the sampling criteria since, according to Weeks’ work (26), the sample size to be obtained in the event of a 95% confidence level and +5% accuracy was calculated to be *n* = 384.

**Table 1:** Demographic characteristics of participants.

Age groups	Males (*N*, %)	Total (*N*)	Age (years)
7-years children	33 (50.00)	66	7.55 ± 0.24
8-years children	45 (47.87)	94	8.46 ± 0.28
9-years children	45 (54.88)	82	9.43 ± 0.28
10-years children	31 (45.59)	68	10.41 ± 0.30
11-years children	52 (53.06)	98	11.50 ± 0.28
Total	496 (50.49)	408	N/A

### Experimental tasks

#### RAN tasks

We employed a Chinese adaptation of RAN (C-RAN) (27) that substituted highly-frequently-used Chinese characters for English letters. The C-RAN paradigm in this study consisted of four tasks: Task N-number (i.e., naming of numbers), Task N-character (i.e., naming of Chinese characters), Task N-object (i.e., naming of objects), and Task N-color (i.e., naming of colors). While, N-object and N-color were non-alphanumeric RAN tasks; N-number and N-character were alphanumeric RAN tasks. For each task, a 5 × 10 matrix of objects was presented, in which each matrix used five repetitions of each of the ten different objects with the order pseudo-randomized.

The subject was situated between 60 and 90 cm away from the 21.5 in. TFT LCD monitor (with 1,920 × 1,080 resolution) displaying the stimuli for each C-RAN task. Eye movements were recorded using a Tobii 4C eye tracker (90 Hz; Tobii Technology AB, Danderyd, Sweden), which was calibrated using a standard 9-point grid. For each RAN task, participants were instructed to name the stimuli (numbers, Chinese characters, colors, or objects) as quickly and accurately as possible in a left-to-right and down fashion.

#### Number cancellation test

As a second experimental task, a number cancellation test (NCT) (28) was used to gauge a participant's attention-related skills. The participant was given a standard B5-sized piece of paper with a list of numbers structured into 26 rows and 40 columns. The participant, who was given a Digital Pen (with an integrated smart mini-camera), was required to find the number “3” (the targeted number) and then draw a circle on it, but ignore all other numbers (distractors), as quickly as possible within 2 min. The Digital Pen's technical advantage was the use of a smart mini-camera, which was designed to measure temporal-spatial features from the perspective of handwriting kinematics, such as pre-movement time (initiating), movement time (moving pen to a stimulus), drawing time (completing a cancellation), circumference of a drawn curve, real-time spatial positions (trajectory) of drawing, and drawing time sequence. It should be noted (28) that temporal-spatial features may outperform traditional NCT measures.

#### Quality control

To ensure the consistency and fidelity of administration of evaluation tools, a senior expert with professional experience more than 8 years carried out measures for all participating kids. The senior expert had training in administration of all tools used in this study.

### Measures

#### Measures of eye movements during RAN

This study took into account six traditional eye-movement measures, including fixation counts, saccade counts, regression counts, average fixation duration, average saccade amplitude, and total time of naming, to assess eye movements during RAN tasks. Two novel measures, namely fixation length fluctuation and saccade amplitude fluctuation, were proposed to indicate the dynamic change of attentional maintenance and switching during RAN. The fixation duration fluctuation was defined as follows:

(1) $$F=\frac{1}{n-1}\overset{n-1}{\underset{i=1}{\sum }}|duration(i+1)-duration(i)|$$

where duration(i) is the time length of the *i*-th fixation.

While, the saccade amplitude fluctuation was defined as follows:

(2) $$F=\frac{1}{n-2}\overset{n-2}{\underset{i=1}{\sum }}|distance(i+1)-distance(i)|$$

where *distance(i)* is the Euclidean distance between the (*i* + 1)-th and *i*-th fixation. In summary, this article will examine eight eye-movement measures (parameters).

#### Measures of a number cancellation test

Several temporal-spatial parameters can be measured with the Digital Pen (which has an integrated smart mini-camera) during NCT (28). In this study, we selected only three parameters (28) to evaluate individuals’ performance during the NCT. Those parameters were defined as follows.

(1) Speed of cognitive processing (SpC) was defined as:
  (3) $$SpC=M\overset{N}{\underset{i=1}{\sum }}R_{i}\;\;\;\;\;\;\;\;\;\;\;\;\;\;\;\;\;\;\;\;\;\;\;\;\;\;\;\;\;\;\;\;\;\;\;\;\;\;\;\;\;\;\;\;\;\;\;\;\;\;\;$$
  where *M* was the amount of numbers in one row (here *M* = 40); *N* was the total number of rows to be circled; $R_{i}=1$ represented the case if any number in the *i*-th row has been circled; and $R_{i}=0$ represented the case if no number in the *i*-th row had been circled.
(2) Selective attention (SA) was defined as:

(4) $$SA=\frac{1}{T}\frac{m-\omega }{m+o}\times \mathrm{SpC}\;\;\;\;\;\;\;\;\;\;\;\;\;\;\;\;\;\;\;\;\;\;\;\;\;\;\;\;\;\;\;\;\;\;\;\;\;\;\;\;\;\;\;\;$$

where *O* was the amount of omitted targets; ω was the number of distractors being circled; and *m* was the total amount of targets that should be circled; *T* was the task time (here *T* = 120); SpC was defined by Equation (1).

(3) Averaged time of circlings (ATC) was defined as:

(5) $$ATC=\frac{1}{n}\times \overset{n}{\underset{i=1}{\sum }}t_{i}\;\;\;\;\;\;\;\;\;\;\;\;\;\;\;\;\;\;\;\;\;\;\;\;\;\;\;\;\;\;\;\;\;\;\;\;\;\;\;\;\;\;\;\;\;\;\;\;$$

where *n* was the amount of numbers being circled; and *t*_*i*_ was the time to circle the *i*-th number.

### Statistical analysis

We performed a three-factor (gender, age, and task-type) ANOVA for each of the eight eye-movement measures, where age (7–11 years) and gender (male vs. female) were inter-group factors; and task-type (Task N-number, Task N-character, Task N-object, and Task N-color) was an intra-group factor. After confirming that our data (i.e., eight eye-movement measures) failed to pass the normality test and variance homogeneity test, we conducted a number of nonparametric ANOVA procedures [i.e., the Aligned Rank Transform (ART) procedures] using ARTool software package (29, 30). Traditional nonparametric statistical tests (like the Kruskal-Wallis test, Mann-Whitney *U* test, Friedman test, or Wilcoxon signed-rank test) are one-way tests and only permit the analysis of one factor at a time, whereas ARTool can be used to analyze multiple factors nonparametrically (29, 30). Additionally, for *post-hoc* multiple comparisons, we utilized the nonparametric Wilcoxon rank-sum test with the “FDR” approach to control the false discovery rate. The effect size was determined by the parameter *r* (low effect: 0.1 ≤ *r* < 0.3; medium effect: 0.3 ≤ *r* < 0.5; efficient response: *r* ≥ 0.5).

Pearson's correlation method was used to calculate the correlation among eight eye-movement measures, as well as the correlation between eight eye-movement features and three parameters of the number cancellation test (28). All statistical analysis above was conducted with R language (version 4.0.2), and the significance level *α* was set at 0.05.

## Results

### General information of participants

The current study investigated a total of 408 children, including 206 males and 202 females. The ratio of males to females was 1.01:1 and the participants were split up into 5 age groups, see Table 1 for detailed information. We verified that there was no significant gender difference ($\chi {}^{2}$ = 1.81, *p* = 0.77).

### Main effects analysis

We performed a three-factor (gender, age, and task-type) ANOVA and *post-hoc* multiple comparisons for each of the eight eye-movement measurements. Figures 1–8 summarized our results, which were listed as follows.

**Figure 1:**
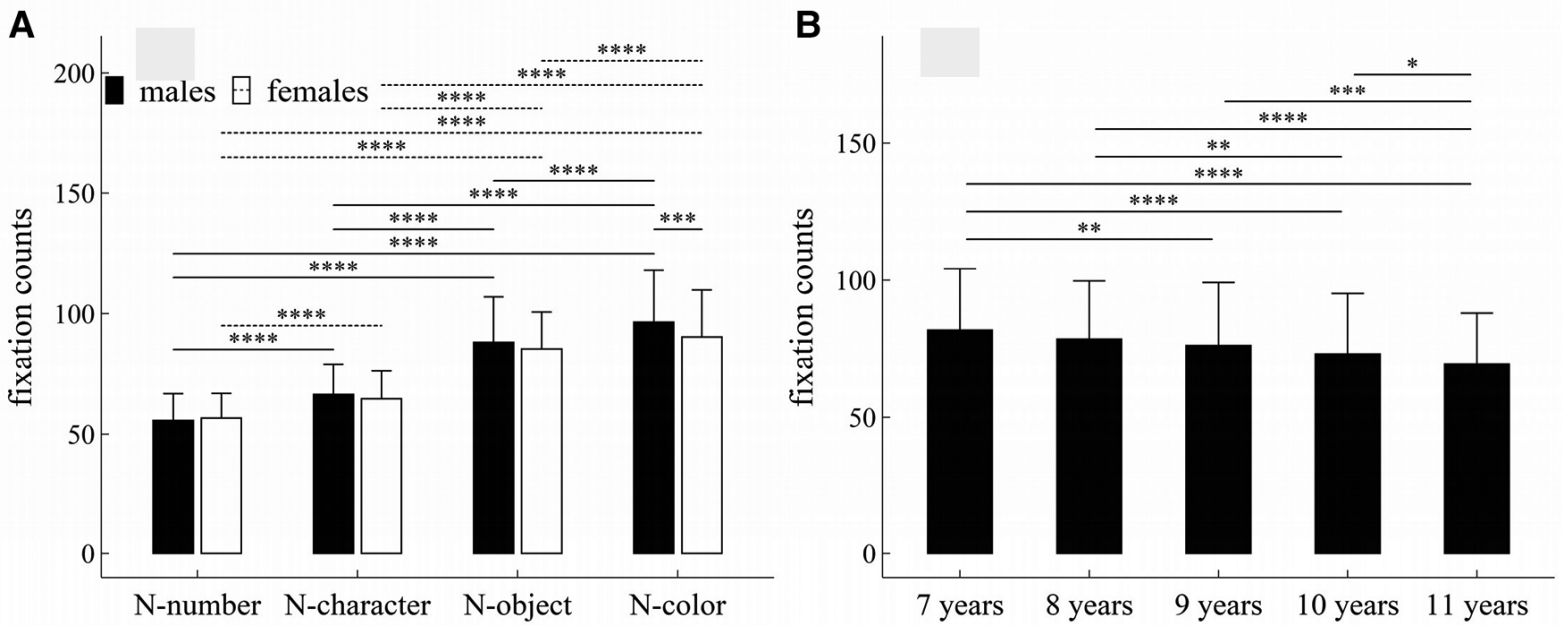
Influence of task-type, gender and age on fixation counts: (**A**) interactive effect of gender and task-type; (**B**) influence of age. *p < 0.05; **p < 0.01; ***p < 0.001; ****p < 1 × 10^−4^.

**Figure 2:**
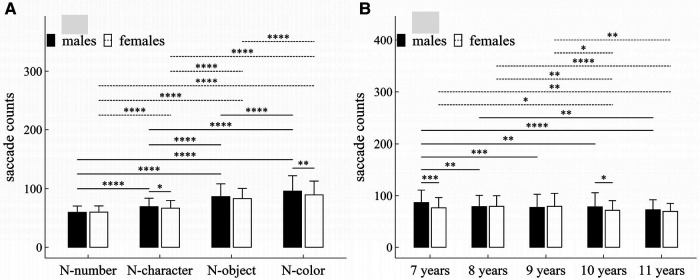
Influence of task-type, gender and age on saccade counts: (**A**) interactive effect of gender and task-type; (**B**) interactive effect of age and task-type. *p < 0.05; **p < 0.01; ***p < 0.001; ****p < 1 × 10^−4^.

**Figure 3:**
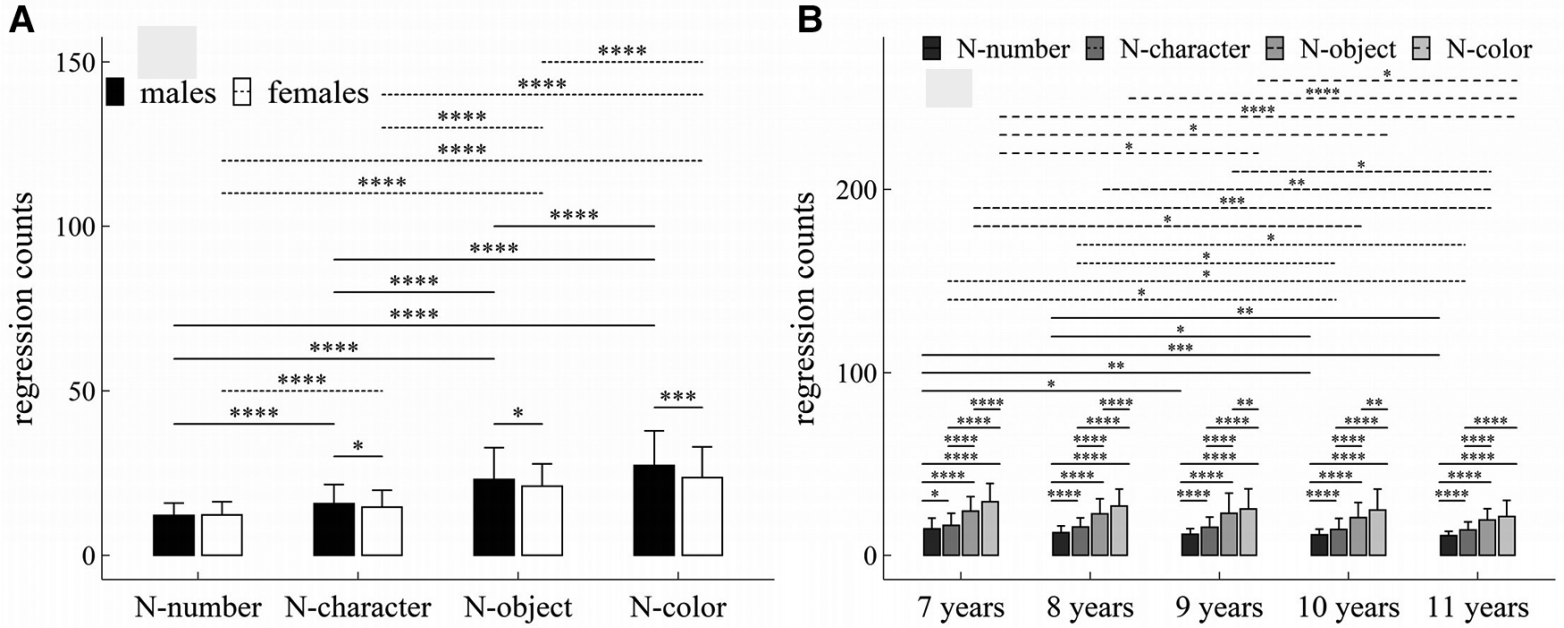
Influence of task-type, gender and age on regression counts: (**A**) interactive effect of gender and task-type; (**B**) interactive effect of age and task-type. *p < 0.05; **p < 0.01; ***p < 0.001; ****p < 1 × 10^−4^.

**Figure 4:**
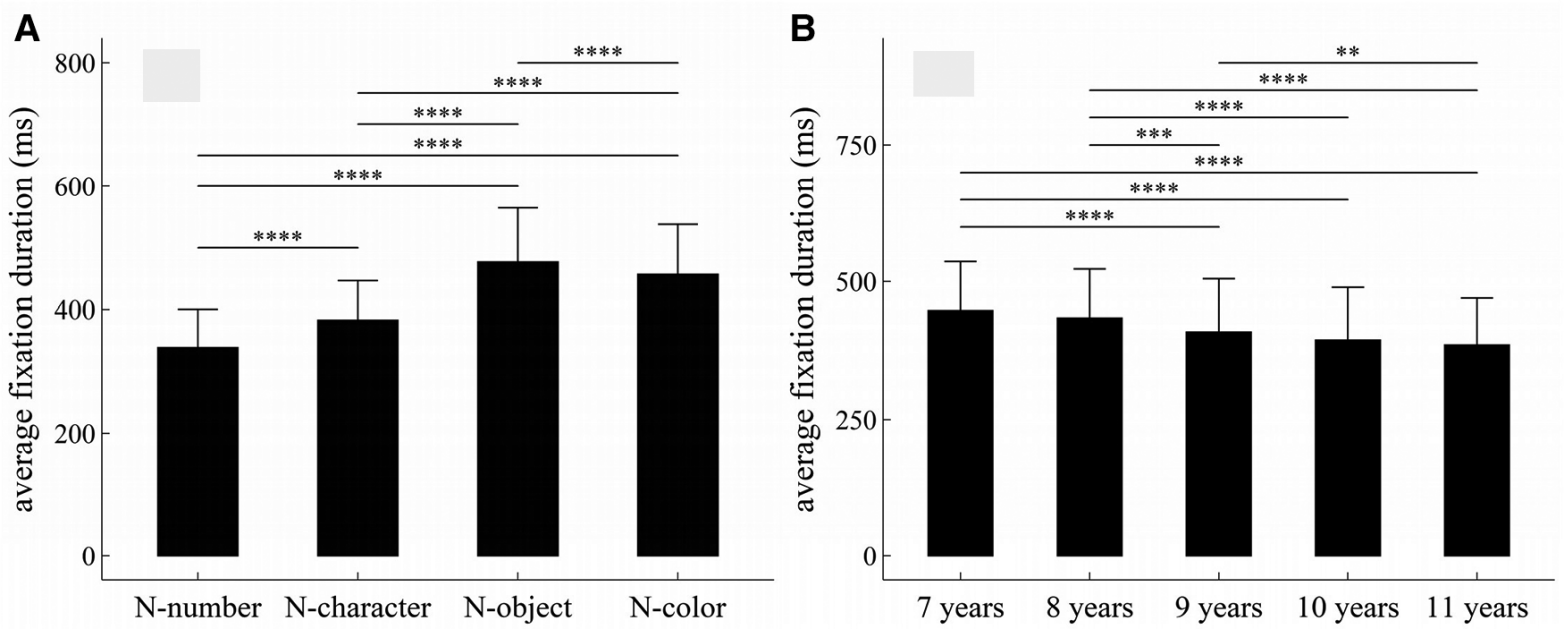
Influence of task-type, gender and age on average fixation duration: (**A**) influence of task-type; (**B**) influence of age. *p < 0.05; **p < 0.01; ***p < 0.001; ****p < 1 × 10^−4^.

**Figure 5:**
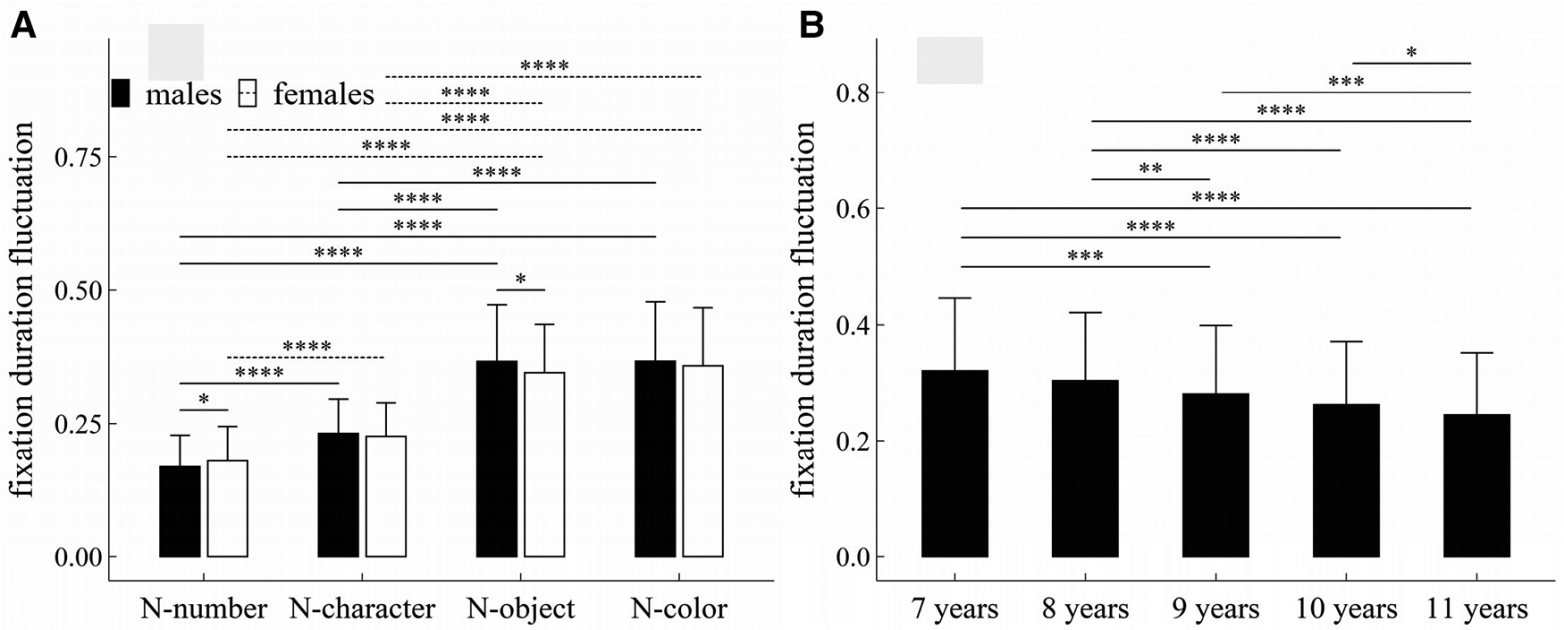
Influence of task-type, gender and age on fixation duration fluctuation: (**A**) interactive effect of gender and task-type; (**B**) influence of age. *p < 0.05; **p < 0.01; ***p < 0.001; ****p < 1 × 10^−4^.

**Figure 6:**
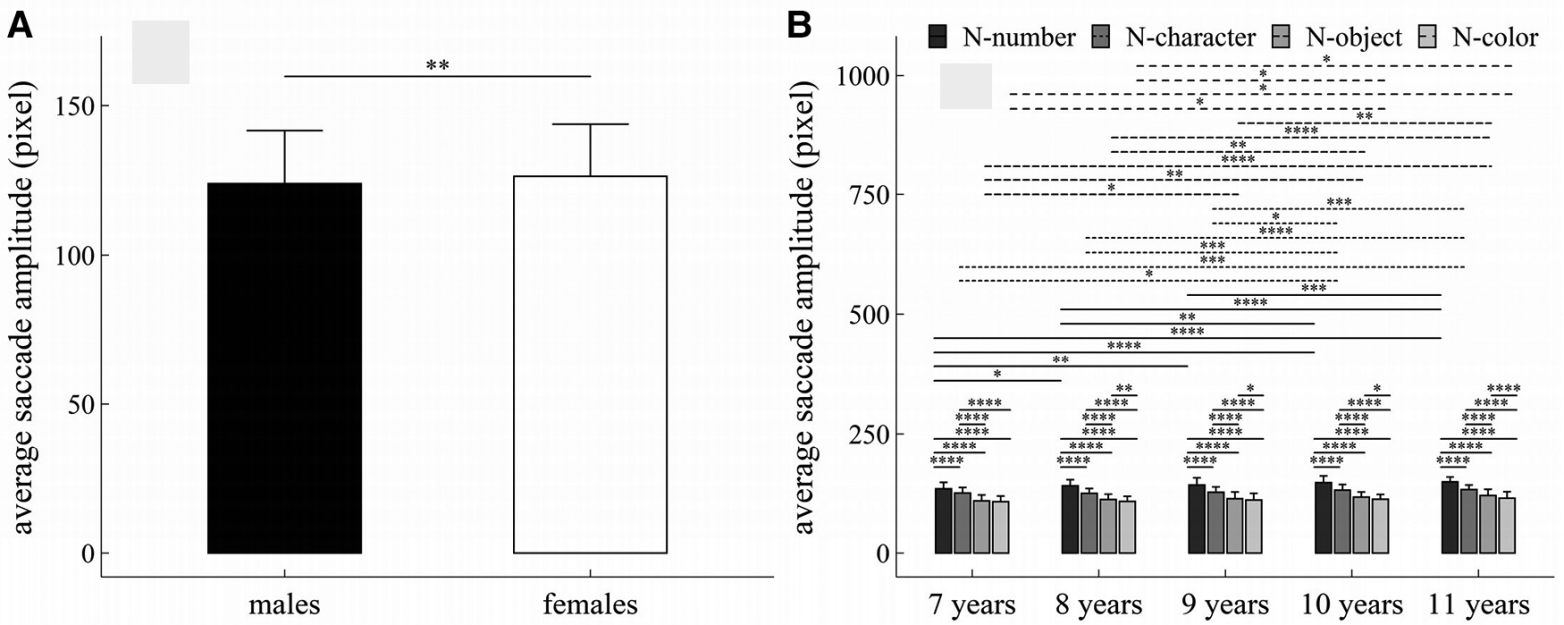
Influence of task-type, gender and age on average saccade amplitude: (**A**) influence of gender; (**B**) interactive effect of age and task-type. *p < 0.05; **p < 0.01; ***p < 0.001; ****p < 0.0001.

**Figure 7:**
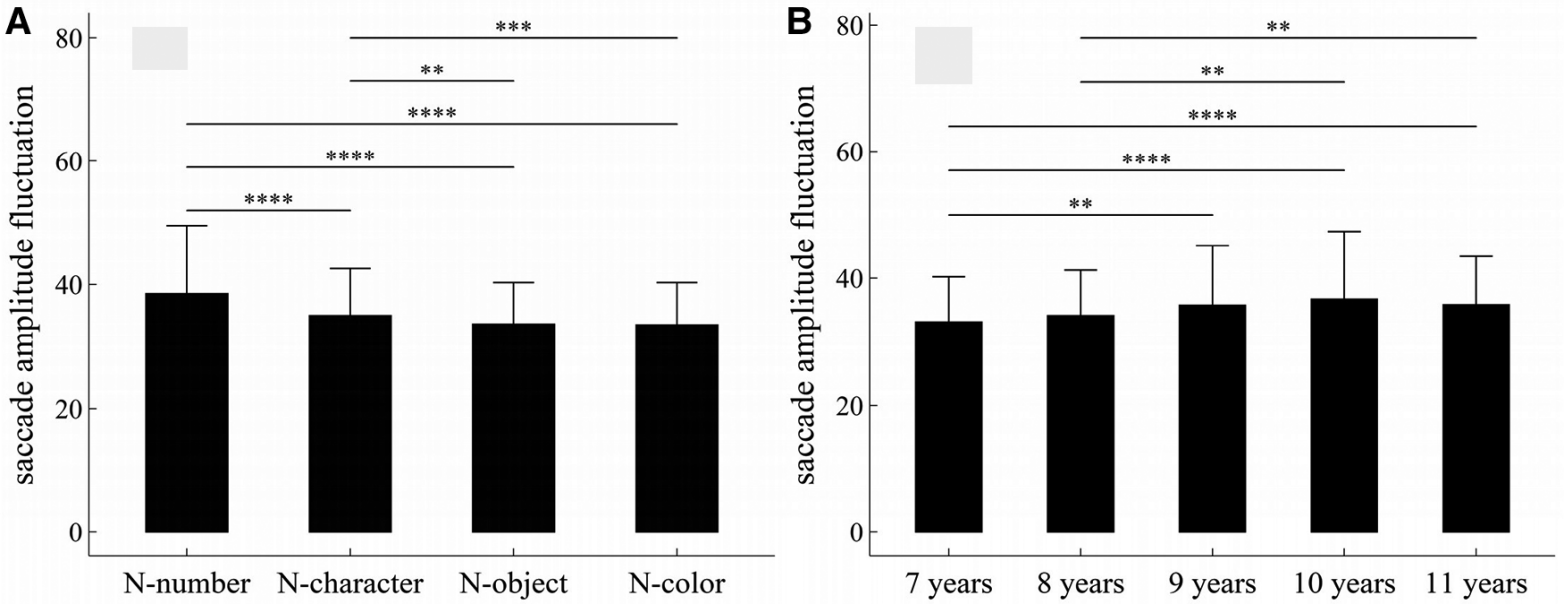
Influence of task-type, gender and age on saccade amplitude fluctuation: (**A**) influence of task-type; (**B**) influence of age. *p < 0.05; **p < 0.01; ***p < 0.001; ****p < 1 × 10^−4^.

**Figure 8:**
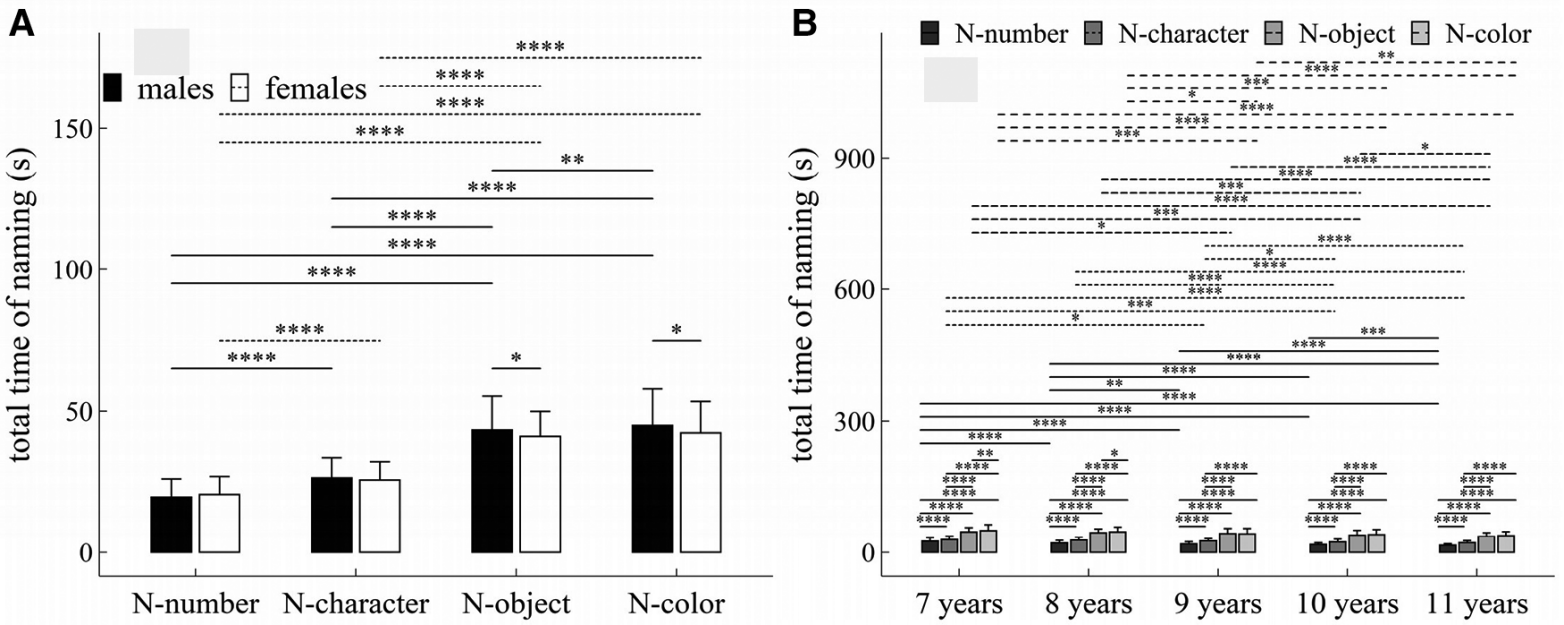
Influence of task-type, gender and age on total time of naming: (**A**) interactive effect of gender and task-type; (**B**) interactive effect of age and task-type. *p < 0.05; **p < 0.01; ***p < 0.001; ****p < 1 × 10^−4^.

#### Fixation counts

##### Main effects

The main effects of task-type, age and gender were significant (Task-type: *F* = 942.32, *p* < 1 × 10^−4^, *η*^2^ = 0.506; Gender: *F* = 6.61, *p* = 0.01, *η*^2^ = 0.009; Age: *F* = 15.15, *p* < 1 × 10^−4^, *η*^2^ = 0.08).

##### Interaction effects

There was a significant interaction effect between gender and task-type (*F* = 6.83, *p* < 1 × 10^−4^, *η*^2^ = 0.007).

##### *Post-hoc* multiple comparisons

Figure 1 summarized the results of *post-hoc* multiple comparisons and indicated that: (1) For males or females (see Figure 1A), task N-number required less fixation counts than other three tasks (*p*’*s* < 1 × 10^−4^, adjusted); task N-character required less fixation counts than tasks N-object and N-color (*p*’*s* < 1 × 10^−4^, adjusted); task N-object required less fixation counts than tasks N-color (*p*’*s* < 1 × 10^−4^, adjusted); (2) As shown in Figure 1A, males required more fixation counts in task N-color than females (*w* = 25032.0, *p* < 0.001, *r* = 0.18), but there was no significant gender difference in other tasks (*p*’*s* > 0.05, adjusted); (3) As shown in Figure 1B, children aged 7 years required more fixation counts than children aged 9–11 years (*p*’*s* < 0.01, adjusted); children aged 8 years required more fixation counts than children aged 10–11 years (*p*’*s* < 0.01, adjusted); children aged 9 or 10 years required more fixation counts than children aged 11 years (*p*’*s* < 0.05, adjusted); but there were no significant differences in fixation counts between children 7 and 8 years, between children 8 and 9 years, and between children 9 and 10 years (*p*’*s* > 0.05, adjusted).

#### Saccade counts

##### Main effects

The main effects of task-type, age and gender were significant (Task-type: *F* = 506.55, *p* < 1 × 10^−4^, *η*^2^ = 0.36; Gender: *F* = 6.61, *p* = 0.01, *η*^2^ = 0.009; Age: *F* = 7.63, *p* < 1 × 10^−4^, *η*^2^ = 0.04).

##### Interaction effects

There were significant interaction effects between gender and task-type (*F* = 4.59, *p* < 0.001, *η*^2^ = 0.005), and between age and gender (*F* = 2.63, *p* = 0.034, *η*^2^ = 0.02).

##### *Post-hoc* multiple comparisons

Figure 2 summarized the results of *post-hoc* multiple comparisons and indicated that: (1) For males or females (see Figure 2A), task N-number required less saccade counts than other three tasks (*p*’*s* < 1 × 10^−4^, adjusted); task N-character required less saccade counts than tasks N-object and N-color (*p*’*s* < 1 × 10^−4^, adjusted); task N-object required less saccade counts than tasks N-color (*p*’*s* < 1 × 10^−4^, adjusted); (2) As shown in Figure 2A, males required more saccade counts in tasks N-character and N-color than females (N-character: *w* = 23145.0, *p* = 0.049, *r* = 0.10; N-color: *w* = 24290.0, *p* = 0.003, *r* = 0.15), but there was no significant gender difference in other tasks (*p*’*s* > 0.05, adjusted); (3) As shown in Figure 2B, males aged 7 years required more saccade counts than males aged 8–11 years (*p*’*s* < 0.01, adjusted); males aged 8 years required more saccade counts than males aged 11 years (*w* = 21976.0, *p* = 0.006, *r* = 0.15, adjusted); but there were no significant differences in saccade counts among males aged 8–10 years, and among males aged 9–11 years (*p*’*s* > 0.05, adjusted); (4) As shown in Figure 2B, females aged 7, 8 or 9 years required more saccade counts than females aged 10–11 years (*p*’*s* < 0.05, adjusted); but there were no significant differences in saccade counts between females aged 7 and 8 years, between females aged 8 and 9 years, and between females aged 10 and 11 years, (*p*’*s* > 0.05, adjusted); (5) As shown in Figure 2B, males aged 7 years required more saccade counts than females aged 7 (*w* = 10905.0, *p* < 0.001, *r* = 0.22); and males aged 10 years required more saccade counts than females aged 10 (*w* = 10592.0, *p* = 0.028, *r* = 0.13).

#### Regression counts

##### Main effects

The main effects of gender, age and task-type were significant (Task-type: *F* = 407.45, *p* < 1 × 10^−4^, *η*^2^ = 0.35; Gender: *F* = 13.27, *p* < 1 × 10^−4^, *η*^2^ = 0.02; Age: *F* = 10.48, *p* < 1 × 10^−4^, *η*^2^ = 0.05).

##### Interaction effects

There were significant interaction effects between age and task-type (*F* = 2.35, *p* = 0.01, *η*^2^ = 0.01), and between gender and task-type (*F* = 7.78, *p* < 1 × 10^−4^, *η*^2^ = 0.01).

##### *Post-hoc* multiple comparisons

Figure 3 summarized the results of *post-hoc* multiple comparisons and indicated that: (1) For males or females (see Figure 3A), task N-number required more regression counts than other three tasks (*p*’*s* < 1 × 10^−4^, adjusted); task N-character required more regression counts than tasks N-object and N-color (*p*’*s* < 1 × 10^−4^, adjusted), task N-object required more regression counts than task N-color (*p*’*s* < 1 × 10^−4^, adjusted); (2) As shown in Figure 3A, males required more regression counts in task N-character (*w* = 23194.5, *p* = 0.044, *r* = 0.10), task N-object (*w* = 23145.5, *p* = 0.049, *r* = 0.097), and task N-color (*w* = 25164.5, *p* < 0.001, *r* = 0.18) than females, but there was no significant gender difference in regression counts in task N-number (*w* = 20148.0, *p* = 0.58, *r* = 0.03); (3) For children aged 7–10 years (see Figure 3B), task N-number required less regression counts than other three tasks (*p*’*s* < 0.05, adjusted); task N-character required less regression counts than tasks N-object and N-color (*p*’*s* < 1 × 10^−4^, adjusted), task N-object required less regression counts than task N-color (*p*’*s* < 0.01, adjusted); (4) For children aged 11 years (see Figure 3B), task N-number required less regression counts than other three tasks (*p*’*s* < 1 × 10^−4^, adjusted); task N-character required less regression counts than tasks N-object and N-color (*p*’*s* < 1 × 10^−4^, adjusted); but there was no significant difference in regression counts between tasks N-object and N-color (*w* = 1593.5, *p* = 0.09, *r* = 0.09); (5) For task N-number (see Figure 3B), children aged 7 years required more regression counts than children aged 9–11 years (*p*’*s* < 0.05, adjusted); children aged 8 years required more regression counts than children aged 10–11 years (*p*’*s* < 0.05, adjusted); but there were no significant differences between children 7 and 8 years, and among children aged 9–11years (*p*’*s* > 0.05, adjusted); (6) For task N-character (see Figure 3B), children aged 7 years required more regression counts than children aged 10–11 years (*p*’*s* < 0.05, adjusted); children aged 8 years required more regression counts than children aged 10–11 years (*p*’*s* < 0.05, adjusted); but there were no significant differences in regression counts between children aged 7 and 8 years, and among children aged 9–11years (*p*’*s* > 0.05, adjusted); (7) For task N-object (see Figure 3B), children aged 7 years required more regression counts than children aged 10–11 years (*p*’*s* < 0.05, adjusted); children aged 8 or 9 years required more regression counts than children aged 11 years (*p*’*s* < 0.05, adjusted); but there were no significant differences in regression counts between children aged 7 and 8 years, between children aged 8 and 9 years, and between children aged 8 and 10 years, and between children aged 10 and 11 years (*p*’*s* > 0.05, adjusted); (8) For task N-color (see Figure 3B), children aged 7 years required more regression counts than children aged 9–11 years (*p*’*s* < 0.05, adjusted); children aged 8 or 9 years required more regression counts than children aged 11 years (*p*’*s* < 0.05, adjusted); but there were no significant differences in regression counts between children aged 7 and 8 years, between children aged 8 and 9 years, and between children aged 10 and 11 years (*p*’*s* > 0.05, adjusted).

#### Average fixation duration

##### Main effects

The main effects of task-type and age were significant (Task-type: *F* = 738.31, *p* < 1 × 10^−4^, *η*^2^ = 0.37; Age: *F* = 15.58, *p* < 1 × 10^−4^, *η*^2^ = 0.096).

##### Interaction effects

There was no significant interaction effect among gender, age and task-type (*p*’*s* > 0.05).

##### *Post-hoc* multiple comparisons

Figure 4 summarized the results of *post-hoc* multiple comparisons and indicated that: (1) As shown in Figure 4A, task N-number required shorter average fixation duration than other three tasks (*p*’*s* < 1 × 10^−4^, adjusted), task N-character required shorter average fixation duration than tasks N-object and N-color (*p*’*s* < 1 × 10^−4^, adjusted), task N-object required longer average fixation duration than task N-color (*w* = 57045, *p* < 1 × 10^−4^, *r* = 0.12, adjusted); (2) As shown in Figure 4B, children aged 7 or 8 years required longer average fixation duration than children aged 9–11 years (*p*’*s* < 0.001, adjusted); children aged 9 years required longer average fixation duration than children aged 11 years (*p*’*s* < 0.01, adjusted); but there were no significant differences in average fixation duration between children 7 and 8 years, between children 9 and 10 years, and between children 10 and 11 years (*p*’*s* > 0.05, adjusted).

#### Fixation duration fluctuation

##### Main effects

The main effects of task-type and age were significant (Task-type: *F* = 902.10, *p* < 1 × 10^−4^, *η*^2^ = 0.495; Age: *F* = 19.71, *p* < 1 × 10^−4^, *η*^2^ = 0.10).

##### Interaction effects

There was a significant interaction effect between gender and task-type (*F* = 4.53, *p* = 0.007, *η*^2^ = 0.005).

##### *Post-hoc* multiple comparisons

Figure 5 summarized the results of *post-hoc* multiple comparisons and indicated that: (1) For males or females (see Figure 5A), task N-number required smaller fixation duration fluctuation than other three tasks (*p*’*s* < 1 × 10^−4^, adjusted), task N-character required smaller fixation duration fluctuation than tasks N-object and N-color (*p*’*s* < 1 × 10^−4^, adjusted), but there were no significant differences in fixation duration fluctuation between task N-object and task N-color (*p*’*s* > 0.05, adjusted); (2) As shown in Figure 5A, males required smaller fixation duration fluctuation in task N-number (*w* = 18399.0, *p* = 0.043, *r* = 0.10), but required larger fixation duration fluctuation in task N-object (*w* = 23553, *p* = 0.021, *r* = 0.114); there were no significant gender difference in tasks N-character and N-color (*p*’*s* > 0.05, adjusted); (3) As shown in Figure 5B, children aged 7 or 8 years required larger fixation duration fluctuation than children aged 9–11 years (*p*’*s* < 0.01, adjusted); children aged 9 years required larger fixation duration fluctuation than children aged 11 years (*w* = 75325.5, *p* < 0.001, *r* = 0.15, adjusted); children aged 10 years required larger fixation duration fluctuation than children aged 11 years (*w* = 58458.5, *p* = 0.043, *r* = 0.08, adjusted); but there were no significant differences in fixation duration fluctuation between children 7 and 8 years, and between children 9 and 10 years (*p*’*s* > 0.05, adjusted).

#### Average saccade amplitude

##### Main effects

The main effects of gender, age and task-type were significant (Task-type: *F* = 1024.49, *p* < 1 × 10^−4^, *η*^2^ = 0.52; Gender: *F* = 9.88, *p* = 0.002, *η*^2^ = 0.014; Age: *F* = 16.29, *p* < 1 × 10^−4^, *η*^2^ = 0.087).

##### Interaction effects

There were significant interaction effects between age and task-type (*F* = 1.96, *p* = 0.03, *η*^2^ = 0.008).

##### *Post-hoc* multiple comparisons

Figure 6 summarized the results of *post-hoc* multiple comparisons and indicated that: (1) As shown in Figure 6A, females required larger average saccade amplitude than males in all RAN tasks (*w* = 304618, *p* = 0.003, *r* = 0.07); (2) For children aged 8–11 years (see Figure 6B), task N-number required larger average saccade amplitude than other three tasks (*p*’*s* < 1 × 10^−4^, adjusted); task N-character required larger average saccade amplitude than tasks N-object and N-color (*p*’*s* < 1 × 10^−4^, adjusted), task N-object required larger average saccade amplitude than task N-color (*p*’*s* < 0.05, adjusted); (3) For children aged 7 years (see Figure 6B), task N-number required larger average saccade amplitude than other three tasks (*p*’*s* < 1 × 10^−4^, adjusted); task N-character required larger average saccade amplitude than tasks N-object and N-color (*p*’*s* < 1 × 10^−4^, adjusted); but there was no significant difference in average saccade amplitude between tasks N-object and N-color (*w* = 1387, *p* = 0.07, *r* = 0.06, adjusted); (4) For task N-number (see Figure 6B), children aged 7 years required smaller average saccade amplitude than children aged 8–11 years (*p*’*s* < 0.05, adjusted); children aged 8 years required smaller average saccade amplitude than children aged 10–11 years (*p*’*s* < 0.01, adjusted); children aged 9 years required smaller average saccade amplitude than children aged 11 years (*w* = 2709, *p* < 0.001, *r* = 0.28, adjusted); but there were no significant differences in average saccade amplitude between children 8 and 9 years, between children 9 and 10 years, and between children 10 and 11 years (*p*’*s* > 0.05, adjusted); (5) For task N-character (see Figure 6B), children aged 7 years required smaller average saccade amplitude than children aged 10–11 years (*p*’*s* < 0.05, adjusted); children aged 8 years required smaller average saccade amplitude than children aged 10–11 years (*p*’*s* < 0.001, adjusted); children aged 9 years required smaller average saccade amplitude than children aged 10–11 years (*p*’*s* < 0.05, adjusted); but there were no significant differences in average saccade amplitude between children 7 and 8 years, between children 8 and 9 years, and between children 10 and 11 years (*p*’*s* > 0.05, adjusted); (6) For task N-object (see Figure 6B), children aged 7 years required smaller average saccade amplitude than children aged 9–11 years (*p*’*s* < 0.05, adjusted); children aged 8 years required smaller average saccade amplitude than children aged 10–11 years (*p*’*s* < 0.01, adjusted); children aged 9 years required smaller average saccade amplitude than children aged 11 years (*w* = 2955, *p* = 0.002, *r* = 0.23, adjusted); but there were no significant differences in average saccade amplitude between children 7 and 8 years, between children 8 and 9 years, and between children 10 and 11 years (*p*’*s* > 0.05, adjusted); (7) For task N-color (see Figure 6B), children aged 7 years required smaller average saccade amplitude than children aged 10–11 years (*p*’*s* < 0.05, adjusted); children aged 8 years required smaller average saccade amplitude than children aged 10–11 years (*p*’*s* < 0.05, adjusted); but there were no significant differences in average saccade amplitude between children 7 and 8 years, between children 8 and 9 years, between children 9 and 10 years, and between children 10 and 11 years (*p*’*s* > 0.05, adjusted).

#### Saccade amplitude fluctuation

##### Main effects

The main effects of task-type and age were significant (Task-type: *F* = 44.90, *p* < 1 × 10^−4^, *η*^2^ = 0.055; Age: *F* = 4.50, *p* = 0.001, *η*^2^ = 0.02).

##### Interaction effects

There was no significant interaction effect among gender, age and task-type (*p*’*s* > 0.05).

##### *Post-hoc* multiple comparisons

Figure 7 summarized the results of *post-hoc* multiple comparisons and indicated that: (1) As shown in Figure 7A, task N-number required larger saccade amplitude fluctuation than other three tasks (*p*’*s* < 1 × 10^−4^, adjusted); task N-character required larger saccade amplitude fluctuation than tasks N-object and N-color (*p*’*s* < 0.01, adjusted); but there was no significant difference in saccade amplitude fluctuation between tasks N-object and N-color (*w* = 43107, *p* = 0.50, *r* = 0.01, adjusted); (2) As shown in Figure 7B, children aged 7 years required smaller saccade amplitude fluctuation than children aged 9–11 years (*p*’*s* < 0.01, adjusted); children aged 8 years required smaller saccade amplitude fluctuation than children aged 10–11 years (*p*’*s* < 0.01, adjusted); but there were no significant differences in saccade amplitude fluctuation between children aged 7 and 8 years, between children aged 8 and 9 years, and among children aged 9–11years (*p*’*s* > 0.05, adjusted).

#### Total time of naming

##### Main effects

The main effects of gender, age and task-type were significant (Task-type: *F* = 1440.33, *p* < 1 × 10^−4^, *η*^2^ = 0.59; Gender: *F* = 4.74, *p* = 0.03, *η*^2^ = 0.007; Age: *F* = 27.82, *p* < 1 × 10^−4^, *η*^2^ = 0.14).

##### Interaction effects

There were significant interaction effects between age and task-type (*F* = 2.37, *p* = 0.01, *η*^2^ = 0.009), and between gender and task-type (*F* = 7.04, *p* < 1 × 10^−4^, *η*^2^ = 0.007).

##### *Post-hoc* multiple comparisons

Figure 8 summarized the results of *post-hoc* multiple comparisons and indicated that: (1) For males (see Figure 8A), task N-number required shorter total time of naming than other three tasks (*p*’*s* < 1 × 10^−4^, adjusted); task N-character required shorter total time of naming than tasks N-object and N-color (*p*’*s* < 1 × 10^−4^, adjusted); and task N-object required shorter total time of naming than task N-color (*p* < 0.01, adjusted); (2) For females (see Figure 8A), task N-number required shorter total time of naming than other three tasks (*p*’*s* < 1 × 10^−4^, adjusted); task N-character required shorter total time of naming than tasks N-object and N-color (*p*’*s* < 1 × 10^−4^, adjusted); but there was no significant difference in total time of naming between tasks N-object and N-color (*w* = 8845.0, *p* = 0.09, *r* = 0.03, adjusted); (3) As shown in Figure 8B, males required longer total time of naming in tasks N-object and N-color than females (*p*’*s* < 0.05), but there were no significant gender difference in total time of naming in tasks N-number and N-character (*p*’*s* > 0.05); (4) For children aged 7–8 years (see Figure 8B), task N-number required shorter total time of naming than other three tasks (*p*’*s* < 1 × 10^−4^, adjusted), task N-character required shorter total time of naming than that tasks N-object and N-color (*p*’*s* < 1 × 10^−4^, adjusted), task N-object required shorter total time of naming task N-color (*p*’*s* < 0.05, adjusted); (5) For children aged 9–11 years (see Figure 8B), task N-number required shorter total time of naming than other three tasks (*p*’*s* < 1 × 10^−4^, adjusted), task N-character required shorter total time of naming than that tasks N-object and N-color (*p*’*s* < 1 × 10^−4^, adjusted), but there was no significant difference in total time of naming between tasks N-object and N-color (*p*’*s* > 0.05, adjusted); (6) For task N-number (see Figure 8B), children aged 7 years required longer total time of naming than children aged 8–11 years (*p*’*s* < 1 × 10^−4^, adjusted); children aged 8 years required longer total time of naming than children aged 9–11 years (*p*’*s* < 0.01, adjusted); children aged 9 or 10 years required longer total time of naming than children aged 11 years (*p*’*s* < 0.001, adjusted); but there was no significant difference between children aged 9 and 10 years (*p*’*s* > 0.05, adjusted); (7) For task N-character (see Figure 8B), children aged 7 years required longer total time of naming than children aged 9–11 years (*p*’*s* < 0.05, adjusted); children aged 8 years required longer total time of naming than children aged 10–11 years (*p*’*s* < 1 × 10^−4^, adjusted); children aged 9 years required longer total time of naming than children aged 10–11 years (*p*’*s* < 0.05, adjusted); but there were no significant differences between children aged 7 and 8 years, between children aged 8 and 9 years, and between children aged 9 and 10 years (*p*’*s* > 0.05, adjusted); (8) For task N-object (see Figure 8B), children aged 7 years required longer total time of naming than children aged 9–11 years (*p*’*s* < 0.05, adjusted); children aged 8 years required longer total time of naming than children aged 10–11 years (*p*’*s* < 0.001, adjusted); children aged 9 or 10 years required longer total time of naming than children aged 11 years (*p*’*s* < 0.05, adjusted); but there were no significant differences between children aged 7 and 8 years, between children aged 8 and 9 years, and between children aged 9 and 10 years (*p*’*s* > 0.05, adjusted); (9) For task N-color (see Figure 8B), children aged 7 years required longer total time of naming than children aged 9–11 years (*p*’*s* < 0.001, adjusted); children aged 8 years required longer total time of naming than children aged 9–11 years (*p*’*s* < 0.05, adjusted); children aged 9 years required longer total time of naming than children aged 11 years (*w* = 5068.5, *p* = 0.004, *r* = 0.22, adjusted); but there were no significant differences between children aged 7 and 8 years, between children aged 9 and 10 years, and between children aged 10 and 11 years (*p*’*s* > 0.05, adjusted).

### Correlation among eye-movement measures

**Figure 9:**
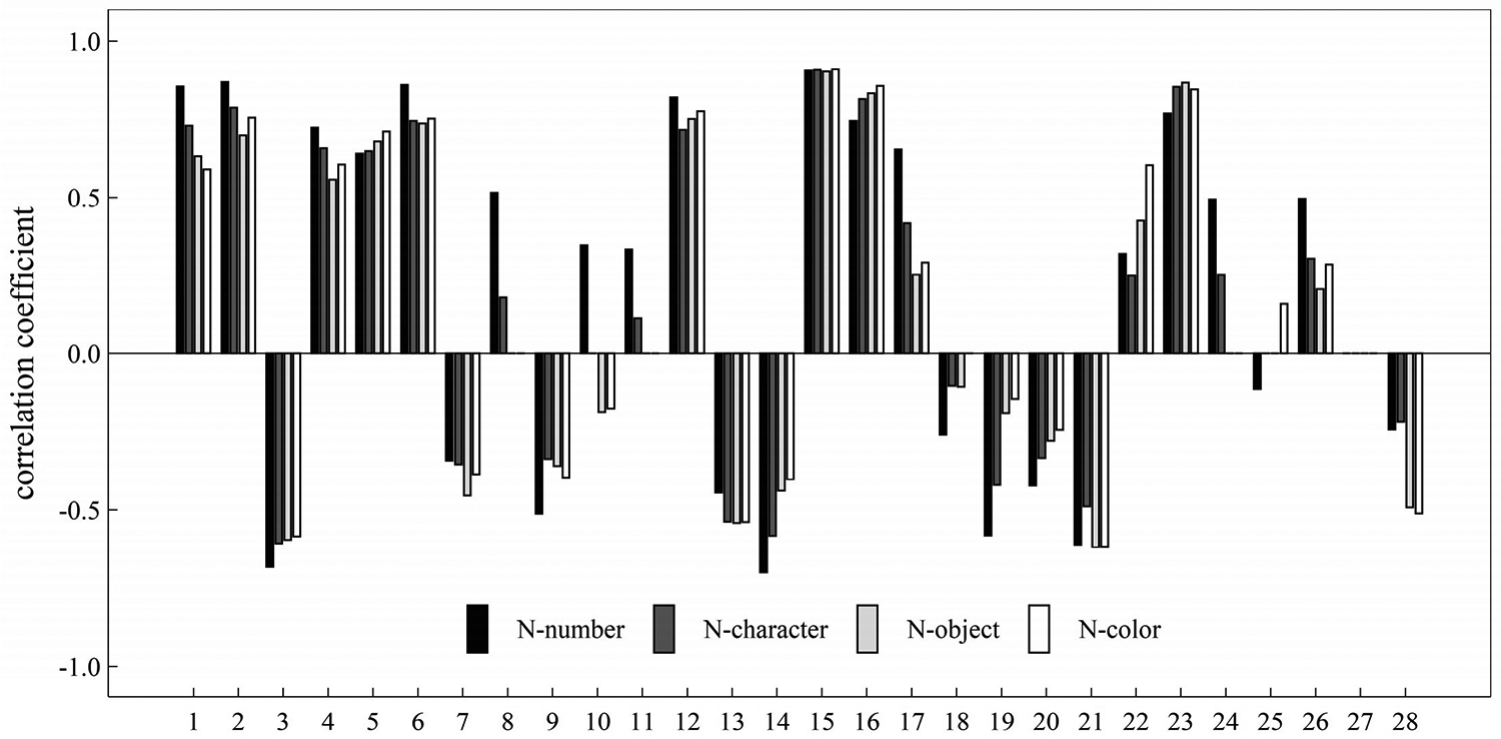
Plot showed correlation coefficients between two eye-movement measures during four tasks, in which the sequence number was defined in Table 2.

If there was a significant correlation between two of the eight eye movement measurements, there were $C_{8}^{2}=28$ possible pairs of two measures, and 28 different correlation coefficients between each pair of two measures. We artificially defined the sequence number for each of the 28 correlation coefficients to better highlight how they differ across the four RAN challenges (see Table 2 for the matching relationship between the sequence number and a correlation coefficient). Figure 9 provided a summary of our findings and displayed the correlation coefficients across four RAN tasks. Pairs having weak correlation in a RAN task were not plotted in Figure 9.

**Table 2:** Sequence number of 28 correlation coefficients between two measures.

No.	Correlation coefficient	No.	Correlation coefficient	No.	Correlation coefficient	No.	Correlation coefficient
1	R (TTN, AFD)	8	R (AFD, FC)	15	R (FC, SC)	22	R (ASA, SAF)
2	R (TTN, FC)	9	R (AFD, ASA)	16	R (FC, RC)	23	R (SC, RC)
3	R (TTN, ASA)	10	R (AFD, SC)	17	R (FC, FDF)	24	R (SC, FDF)
4	R (TTN, SC)	11	R (AFD, RC)	18	R (FC, SAF)	25	R (SC, SAF)
5	R (TTN, RC)	12	R (AFD, FDF)	19	R (ASA, SC),	26	R (RC, FDF)
6	R (TTN, FDF)	13	R (AFD, SAF)	20	R (ASA, RC)	27	R (RC, SAF)
7	R (TTN, SAF)	14	R (FC, ASA)	21	R (ASA, FDF)	28	R (FDF, SAF)

R(*x,y*) is the correlation coefficient between measures *x* and *y*; FC, fixation counts; SC, saccade counts; RC, regression counts; AFD, average fixation duration; FDF, fixation duration fluctuation; ASA, average saccade amplitude; SAF, saccade amplitude fluctuation; TTN, total time of naming.

It can be easily seen from Figure 9 that: (i) There were significant positive correlation in 12 pairs of measures across all RAN tasks; (ii) There were significant negative correlation in 10 pairs of measures across all RAN tasks; (iii) There were no significant correlation in one pair (No. 27, corresponding to the pair of regression counts and saccade amplitude fluctuation) across all RAN tasks; and (iv) There were significant correlation in two pairs (Nos. 8 and 11, corresponding to the pair of average fixation duration and fixation counts, and the pair of average fixation duration and regression counts, respectively) in tasks N-number and N-character, but there were no significant correlation in other tasks.

As noted, the tasks N-number and N-character were alphanumeric RAN tasks, whereas the tasks N-object and N-color were non-alphanumeric ones. It would be interesting to see if there were any correlation differences between alphanumeric and non-alphanumeric RAN tasks. Our findings (see Figure 9) showed that while the correlations between 6 pairs of measures (i.e., Nos. 5, 7, 16, 21, and 28 in Table 2) in alphanumeric RAN tasks may be higher than those in non-alphanumeric RAN tasks, the correlations between 12 pairs of measures (i.e., Nos. 1, 2, 3, 4, 8, 11, 14, 17, 18, 19, and 26 in Table 2) may be lower in alphanumeric RAN tasks than in non-alphanumeric RAN tasks.

Some important correlations between pairs of measures were listed as follows: (i) The correlations (corresponding to Nos. 1, 2, 4, 5 and 6 in Figure 9) between total time of naming and five measures were positive (*r* between 0.56 and 0.87); the correlation (corresponding to No. 3 Figure 9) between total time of naming and one measures was negative (*r* between −0.68 and −0.58); and the correlation (corresponding to No. 7 in Figure 9) between total time of naming and saccade amplitude fluctuation (*r* between −0.45 and −0.34); (ii) The correlations (corresponding to Nos. 15, 16 and 23 in Figure 9) among three count-related measures (i.e., fixation counts, saccade counts, regression counts) were significantly positive (*r* between 0.74 and 0.91); (iii) The correlation (corresponding to No. 28 in Figure 9) between two fluctuation-related measures (i.e., fixation duration fluctuation, saccade amplitude fluctuation) was significantly negative in all tasks; and the correlation coefficients in alphanumeric RAN tasks (*r* = −0.24 and *r* = −0.22) were higher than that in non-alphanumeric tasks (*r* = −0.49 and *r* = −0.5); (iv) The correlation (corresponding to No. 12 in Figure 9) between two fixation-duration-related measures (i.e., average fixation duration and fixation duration fluctuation) was significantly positive in all tasks (*r* between 0.72 and 0.82); and the correlation coefficient in task N-number was higher than other tasks; and (v) The correlation (corresponding to No. 22 in Figure 9) between two saccade-amplitude-related measures (i.e., average saccade amplitude and saccade amplitude fluctuation) was significantly positive in all tasks (*r* between 0.25 and 0.60); and the correlation coefficients between them in alphanumeric RAN tasks (*r* = 0.32 and *r* = 0.25) were lower than that in non-alphanumeric counterparts (*r* = 0.42 and *r* = 0.60).

### Correlation between eye movements and attention-related skills

We asked the participating kids to complete the RAN tasks and the NCT test (28) together in order to demonstrate the relationship between RAN skills and attention-related abilities. Eight eye-movement measures were selected for RAN tasks, while three attention-related skills indicated for the NCT test were derived using Equations (3–5). The relationship between the measures of the two tasks was then examined. Tables 3–6 summarized our results and showed that: (i) Significant correlations were found between three attention-related skills and eight eye-movement measures (*p*’*s* < 0.05); (ii) In general, two parameters (i.e., speed of cognitive processing and selective attention) significantly correlated negatively with six eye-movement measures (i.e., fixation counts, saccade counts, regression counts, average fixation duration, fixation duration fluctuation, and total time of naming) (*p*’*s* < 0.05), but significantly correlated positively with two other eye-movement measures (i.e., average saccade amplitude and saccade amplitude fluctuation) (*p*’*s* < 0.05); (iii) Basically, averaged time of circlings significantly negatively correlated with two eye-movement measures (i.e., average saccade amplitude and saccade amplitude fluctuation) (*p*’*s* < 0.05), but significantly positively correlated with six eye-movement measures (i.e., fixation counts, saccade counts, regression counts, average fixation duration, fixation duration fluctuation, total time of naming) (*p*’*s* < 0.05).

**Table 3:** Correlation between number-cancellation-related skills and eye-movement measures in task N-naming.

Eye-movement measures	Number cancellation test		
	Speed of cognitive processing	Selective attention	Averaged time of circlings
Fixation counts	−0.25****	−0.26****	0.19***
Saccade counts	−0.19***	−0.18***	0.14**
Regression counts	−0.24****	−0.26****	0.18***
Average fixation duration	−0.22****	−0.28****	0.17****
Fixation duration fluctuation	−0.28****	−0.33****	0.27****
Average saccade amplitude	0.20****	0.27****	−0.18***
Saccade amplitude fluctuation	0.09	0.11*	−0.11*
Total time of naming	−0.34****	−0.38****	0.25****

* *p* < 0.05.

** *p* < 0.01.

*** *p* < 0.001.

**** *p* < 0.0001.

## Discussion

RAN tests have been utilized extensively in the examination of numerous cognitive and neurobiological disorders (13–19), as well as in studies on reading behavior and child development in healthy children (2–8). This article aimed to offer some new perspectives on RAN understanding by the eye tracking method, which is able to capture the visual and cognitive characteristics of RAN. To resolve issues with earlier research, this article involved measures of eye movements during RAN tests for Chinese children aged 7–11 years. This study's primary goal was to determine how age, gender, and task type affected measures of eye movements made by Chinese children aged 7–11 during RAN. Eight parameters (including two fluctuation parameters proposed in this article) were designed to measure eye movements.

First of all, our findings showed that all eye-movement measures had the main effects of task-type and age, but only five of them (i.e., fixation counts, saccade counts, regression counts, average saccade amplitude, total time of naming) had the main effect of gender. Additionally, three measures (i.e., saccade counts, regression counts, and total time of naming) had interaction effects between task-type and gender, and between age and gender; two measures (i.e., fixation counts and fixation duration fluctuation) had an interaction effect between gender and task-type, only; one measure (i.e., average saccade amplitude) had an interaction effect between age and gender, only.

Our results confirm that almost all eye-movement measures initially develop quickly before the age of 9, and then enter a relatively sluggish development phase without a ceiling or floor impact for kids between the ages of 7 and 11. In particular, children aged 7–11 years can be roughly separated into three stages for the development of RAN-related skills: 7–8 years old, 9–10 years old, and 11 years old. This is in line with the widely-used three-stage model in Chinese educational practices, where the low stage corresponds to Grades 1–2 (corresponding to children aged 7–8), the middle stage corresponds to Grades 3–4 (corresponding to children aged 9–10), and the high stage corresponds to Grades 5–6 (corresponding to children aged 11–12 years old).

It is well established that females have a faster cognitive and social development up to the end of adolescence than males of the same age. Our results can likewise be used to support this conclusion. Indeed, we demonstrated that females outperformed males in RAN tasks as measured by five eye-movement parameters: fixation counts, saccade counts, regression counts, average saccade amplitude, and total time of naming. Additionally, the fixation duration fluctuation in tasks N-number and N-object was significantly different between males and females, as shown in Figure 5A. We also discovered that there was no gender difference in saccade amplitude fluctuation. Furthermore, almost all measures (except average fixation duration) had an interaction effect between gender and task-type, or between gender and age. These findings might offer some new perspectives on how gender affects children's cognitive development and neurological and psychiatric disorders connected to RAN.

RAN tasks can generally be grouped into two categories, i.e., alphanumeric and non-alphanumeric RAN tasks. Naming of numbers, letters, words, or Chinese characters are examples of alphanumeric RAN tasks, whereas naming of colors or objects are examples of non-alphanumeric RAN tasks. Alphanumeric RAN might necessitate primarily phonological processing because the associated linguistic codes of these stimuli are easily available at the surface level (31). While non-alphanumeric RAN appears to necessitate extra steps and need conceptual processing to establish meaning and then the appropriate name code, before phonological processing results in articulating a response (31). This suggests that non-alphanumeric RAN is more complex than alphanumeric RAN and hence generally takes more mental effort. This inference may be supported by our findings that in comparison to alphanumeric RAN tasks, non-alphanumeric RAN tasks generally required more fixation counts, saccade counts, regression counts, smaller average saccade amplitude, smaller fixation duration fluctuation and saccade amplitude fluctuation, and longer average fixation duration and total time of naming. This suggests that eight parameters used in this article may inherit key features of eye movements during RAN tasks, and more importantly, may be able to distinguish between alphanumeric and non-alphanumeric RAN.

The fluctuation, defined as the sum of absolute first-order differences in Equation (1) or (2), is taken from an earlier work (32), which can be applied to measure the oscillation, variability, and unpredictability of time series in a variety of nonlinear physical systems. According to the work of Paulson (33), eye movement can be considered as a self-similar nonlinear dynamic process, and thus can be measured using the fluctuation. The eye-movement changes associated with age or task-type may be observed by both fluctuation parameters, as shown in Figures 5, 7. Additionally, the gender differences in tasks N-number and N-object may be reflected in the fixation duration fluctuation, but not in the saccade amplitude fluctuation.

Rather than emphasizing the superiority of the fluctuation definition, this study attempted to employ fluctuation as a supplement to the original measures of eye movements. In fact, we also examined how well the standard deviation performed as a definition of fluctuation and discovered that it had a strong correlation (*r*’s ≥ 0.85) with our technique (i.e., the sum of absolute first-order differences) for all RAN tasks. It can be easily seen from Figure 9 that the correlations among eight measures of eye movements may be positive, negative and null. Some correlations between two measures may decrease with the task complexity, but some of them may increase with the task complexity. While, two pairs of measures had significant correlations in tasks N-number and N-character, only, but not in other tasks. The principle of assessing total time of naming remains the same, even though it was measured in this study using an eye tracking method that differed from the traditional behavioral methodology. It should be noted that total time of naming was well-documented in traditional RAN measures. It was fascinating to see if there were any relationships between total time of naming and other RAN measures. Our findings showed that there were highly correlated between total time of naming and other six measures (|*r*| between 0.56 and 0.87); and there was moderately correlated between total time of naming and saccade amplitude fluctuation (*r* between −0.45 and −0.34). This partially supports the effectiveness and feasibility of the eye-movement measures suggested in the current article.

The correlations among three count-related measures (i.e., fixation counts, saccade counts, regression counts) were significantly positive (*r* between 0.74 and 0.91). This implies that internal consistency among three count-related measures was high. Additionally, the correlation between two fixation-duration-related measures (i.e., average fixation duration and fixation duration fluctuation) was highly positive in all tasks (*r* between 0.72 and 0.82).

Findings (see Tables 4–6) showed that there were moderate correlations between eight eye-movement measures and three attention-related skills (*p*’*s* < 0.05). This supports the weak correlation but strong dependence between RAN and NCT tasks. The NCT was applied to measure attention-related abilities, which involved cognitive skills in selective and sustained attention, motor inhibition, visuospatial search, planning, organizing, psychomotor speed, intact visual-perception abilities, fine motor coordination, and sensory motor integration (28). On the other hand, both RAN and NCT might share several visual and cognitive neural circuits because they both need a similar “visual scanning” processing. Additionally, NCT and RAN are associated with “writing” and “reading”, respectively. Hence, it is hypothesized that RAN, in combination with NCT, may bring some new insights into the understanding of developmental dyslexia and learning disabilities (34).

**Table 4:** Correlation between number-cancellation-related skills and eye-movement measures in task N-character.

Eye-movement measures	Number cancellation test		
	Speed of cognitive processing	Selective attention	Averaged time of circlings
Fixation counts	−0.17***	−0.23****	0.16***
Saccade counts	−0.10	−0.16**	0.09
Regression counts	−0.10*	−0.16**	0.14**
Average fixation duration	−0.34****	−0.37****	0.28****
Fixation duration fluctuation	−0.31****	−0.36****	0.31****
Average saccade amplitude	0.32****	0.36****	−0.27****
Saccade amplitude fluctuation	0.13*	0.10*	−0.09
Total time of naming	−0.32****	−0.39****	0.30****

*p<0.05; **p<0.01; ***p<0.001; ****p<0.0001.

**Table 5:** Correlation between number-cancellation-related skills and eye-movement measures in task N-object.

Eye-movement measures	Number cancellation test		
	Speed of cognitive processing	Selective attention	Averaged time of circlings
Fixation counts	−0.26****	−0.26****	0.19***
Saccade counts	−0.19***	−0.18***	0.14**
Regression counts	−0.24*	−0.26****	0.19***
Average fixation duration	−0.22****	−0.28****	0.17***
Fixation duration fluctuation	−0.26****	−0.32****	0.15**
Average saccade amplitude	0.20****	0.27****	−0.18***
Saccade amplitude fluctuation	0.13**	0.19***	−0.09
Total time of naming	−0.34****	−0.38****	0.25****

*p<0.05; **p<0.01; ***p<0.001; ****p<0.0001.

**Table 6:** Correlation between number-cancellation-related skills and eye-movement measures in task N-color.

Eye-movement measures	Number cancellation test		
	Speed of cognitive processing	Selective attention	Averaged time of circlings
Fixation counts	0.20****	−0.24****	0.17***
Saccade counts	−0.14**	−0.19***	0.10*
Regression counts	−0.21****	−0.28****	0.14**
Average fixation duration	−0.23****	−0.26****	0.18***
Fixation duration fluctuation	−0.23****	−0.27****	0.19***
Average saccade amplitude	0.17***	0.20****	−0.16**
Saccade amplitude fluctuation	0.10*	0.11*	−0.13*
Total time of naming	−0.30****	−0.36****	0.24****

*p<0.05; **p<0.01; ***p<0.001; ****p<0.0001.

It is natural to adopt a Chinese adaptation of RAN in the understanding of developmental dyslexia in Chinese. The difference between both the original RAN and Chinese version is due to the features of Chinese characters: (i) Chinese characters not only have shape and sound attributes like English letters, but also represent meaning; (ii) Chinese characters have no clear form-to-sound conversion rules, so readers need to remember the pronunciation of Chinese characters; and (iii) The visual complexity of Chinese characters are much higher than that of English letters. Consequently, compared with the original RAN, the Chinese adaptation may have higher cognitive complexity, and thus activate a wider range of brain regions (35, 36). To extend the application of RAN to developmental dyslexia in Chinese, we suggested a Chinese adaptation of RAN (i.e., the C-RAN) by substituting Chinese characters (highly-frequently used) for English letters. We expect that the C-RAN should be more suitable in the evaluation of developmental dyslexia in Chinese than the original RAN.

Future study will explore a few issues. First, we will explore gender differences in eye-movement measures taken during RAN throughout the lifespan (especially for children older than 11-years). Second, we will examine the possibility that the apparent gender disparities in RAN-related neurological and mental disorders are due to age-related changes in RAN skills. Third, we will detect whether the diagnostic criteria for RAN-related neurological and psychiatric disorders might be biased or poorly specified for one gender and/or grade group. Finally, we will seek a new definition of fluctuation with increased performance.

## Conclusion

This article investigated eight measures of eye movements during RAN tests for Chinese children aged 7–11 years. First of all, we showed that all eye-movement measures had the main effects of task-type and age, but only five of them had the main effect of gender, with interaction effect between task-type and gender or/and between age and gender. Second, we found that almost all eye-movement measures initially developed quickly before the age of 9, and then entered a relatively sluggish development phase. Third, we confirmed that non-alphanumeric RAN tasks generally required higher mental load than alphanumeric ones. Fourth, we showed that there were significant correlations between total time of naming and other eye-movement measures. Finally, we found significant relationships between eight eye-movement measures and three attention-related skills. Because eye tracking is a fundamental tool in psychological research, the technique suggested has the potential to be used in a wide range of applications (e.g., developmental dyslexia).

## Data Availability

The raw data supporting the conclusions of this article will be made available by the authors, without undue reservation.
